# Acceleration of lipid reproduction by emergence of microscopic motion

**DOI:** 10.1038/s41467-021-23022-1

**Published:** 2021-05-19

**Authors:** Dhanya Babu, Robert J. H. Scanes, Rémi Plamont, Alexander Ryabchun, Federico Lancia, Tibor Kudernac, Stephen P. Fletcher, Nathalie Katsonis

**Affiliations:** 1 Stratingh Institute for Chemistry, University of Groningen, Groningen, The Netherlands; 2 Department of Chemistry, Chemistry Research Laboratory, University of Oxford, Oxford, UK

**Keywords:** Molecular biophysics, Origin of life, Self-assembly

## Abstract

Self-reproducing molecules abound in nature where they support growth and motion of living systems. In artificial settings, chemical reactions can also show complex kinetics of reproduction, however integrating self-reproducing molecules into larger chemical systems remains a challenge towards achieving higher order functionality. Here, we show that self-reproducing lipids can initiate, sustain and accelerate the movement of octanol droplets in water. Reciprocally, the chemotactic movement of the octanol droplets increases the rate of lipid reproduction substantially. Reciprocal coupling between bond-forming chemistry and droplet motility is thus established as an effect of the interplay between molecular-scale events (the self-reproduction of lipid molecules) and microscopic events (the chemotactic movement of the droplets). This coupling between molecular chemistry and microscopic motility offers alternative means of performing work and catalysis in micro-heterogeneous environments.

## Abstract (web summary)

Artificial molecular systems can show complex kinetics of reproduction, however their integration into larger ensembles remains a challenge towards evolving higher order functionality. Here authors use show that self-reproducing lipids can initiate and accelerate octanol droplet movement and that reciprocally chemotactic movement of these droplets increases the rate of lipid reproduction substantially.

## Introduction

Molecules which can facilitate their own formation or “make themselves”^1–4^ have likely played a role in the emergence of living systems^5–8^. Next to the ability to reproduce, the ability to move with a purpose has also been a hallmark of life from the earliest times^9,10^. Chemotaxis, movement in response to a chemical signal^11^, enables the guiding of sperm to ova,^12,13^ the directed migration of neutrophils to inflammation^14^ and other vital processes. Despite molecular self-reproduction and chemotactic motion being both ubiquitous features of life, the intricacies of their interplay remain mostly unknown, partly because the mechanisms which are involved require coupling between chemical processes that occur at the molecular level, with physical processes that occur at the microscopic length scale.

In this context, the self-reproduction of simple models of membranes (e.g. micelles and vesicles) has been investigated^15^, and artificial self-reproducing systems have been developed where hydrophobic molecules react slowly with molecules dissolved in water^16–18^. The lipidic products of these chemical reactions self-assemble into supramolecular aggregates that aid material transfer. Significantly, the reaction products facilitate their own formation, a process known as physical autocatalysis—a product-induced increase in reaction rate that involves molecular self-assembly^19–23^.

Allied chemical autocatalytic systems have led to emergent behavior at the supramolecular level^18,24–26^. However, combining molecular self-reproduction and functional behavior at larger length scales remains a current challenge, mostly because we still lack ultimate control over all the length scales of molecular self-assembly. Here, we employ chemo-motile coupling as a strategy to bridge lipid reproduction with higher order functionality. We show that chemotactic movement of oil droplets emerges from lipid reproduction, and that this motile behavior feeds back into the system, so that overall the rate of lipid replication is enhanced further.

**Fig. 1:**
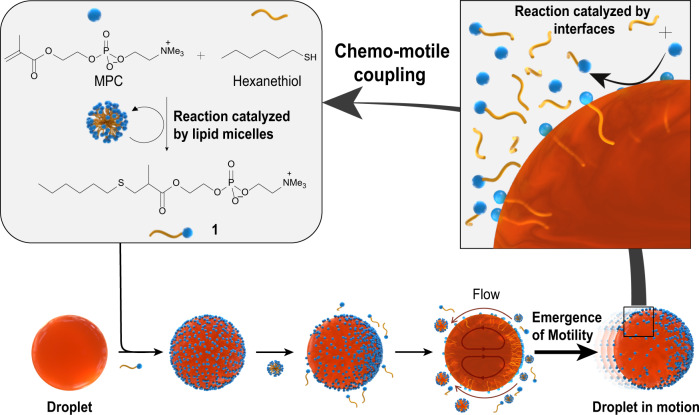
Mechanisms supporting the motion of microscopic droplets in a lipid system. The Michael reaction between an emulsion of 1-hexanethiol and water-soluble 2-methacryloyloxyethyl phosphorylcholine (MPC) forms lipid **1**, which self-assembles to form micelles in water. Once the concentration of **1** reaches the critical propulsion concentration (CPC), micelles filled with oil start forming in the vicinity of the lipid-covered oil droplet, symmetry is broken, and at this point the droplet starts moving towards micelle-rich areas. In return, the rate of lipid self-reproduction is increased.

We have used a Michael reaction between an emulsion of 1-hexanethiol and water-soluble 2-methacryloyloxyethyl phosphorylcholine (MPC) forming lipid **1**, which self-assembles into micelles^27^. Microscopic droplets of octanol are added to this reaction medium. Once the concentration of **1** reaches a critical propulsion concentration (CPC), the lipid-covered octanol droplets start moving towards areas that are rich in 1-hexanethiol. Chemo-motile coupling subsequently develops between lipid reproduction and droplet motion: the lipids self-assemble into micelles that make oil droplets move and, in return, the chemotactic movement of the droplets increases the rate of lipid self-reproduction (Fig. 1).

## Results

### Emergence of droplet motility in a self-reproducing lipid system

The system at the heart of our investigation features a bond-forming reaction between a thiol nucleophile in an oil-in-water emulsion, and a water-soluble electrophile (MPC), to form lipid **1** (Fig. 1 and Supplementary Figure 1). In this process, the lipid aggregates into micelles that facilitate the coupling of phase-separated 1-hexanethiol and MPC, and hence the formation of lipids enhances their own formation^21,28^. Where the bond-forming reaction specifically occurs has been the subject of debate in the context of micellar autocatalysis^16,17^. Independently of which mechanism predominates, lipids are indeed known to affect physical function across length scales, by altering interfacial properties.

In the presence of lipid micelles, microscopic oil droplets move in water autonomously^29–34^, and their motion can be initiated by chemical reactions^35–37^. Such droplets are found to propel chemotactically towards regions of higher micelle concentration^38,39^. Based on the fact that lipids can support surface tension-driven propulsion, we envisioned that an environment favorable to motility could emerge from lipid reproduction.

In our experiments, motility is imparted to microscopic droplets of octanol, and we verified that octanol itself does not react with MPC (Supplementary Fig. 2). The octanol droplets were produced in a microfluidic chip (Supplementary Movie 1) with a narrow polydispersity (Supplementary Fig. 3), and were subsequently added to a chamber containing a freshly prepared mixture of 1-hexanethiol and MPC. Octanol droplets do not maintain a very narrow size distribution once introduced in the aqueous reaction medium, because originally there are no surfactants in this medium and therefore the droplets are not stable. Typically for droplets of 150 µm in diameter, once introduced in the reaction medium, the range becomes [130–180 µm]. The droplets shrink ~10% in diameter in the timeframe of our experiments.

After a lag period during which no motion is observed, the oil droplets start moving as the lipid concentration becomes sufficiently large (Supplementary Movie 2). When either MPC or thiol are absent from the system, motion of octanol droplets was never observed. Thiol droplets that were formed in the reaction mixture did never move, under any conditions.

**Fig. 2:**
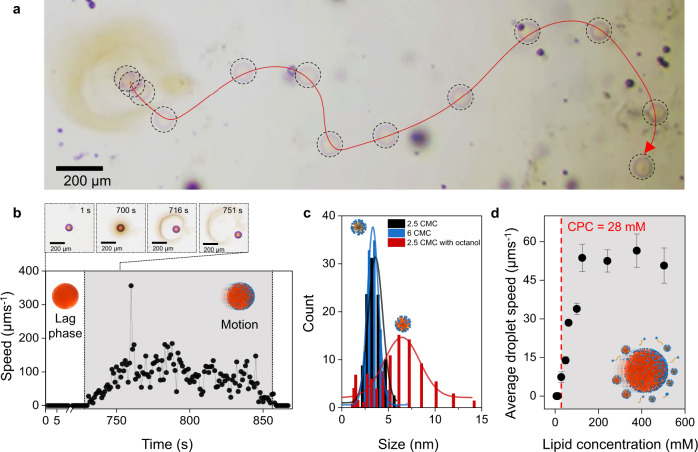
Emergence of droplet motility from lipid reproduction. **a** Trajectory of an octanol droplet tracked over 80 s (Supplementary Movie 3). All octanol droplets moved in these experimental conditions. **b** Speed of an octanol droplet over time (individual trace, diameter of the droplet is 100 µm). The appearance and expansion of a corona occurred during the lag phase for all studied droplets. **c** Dynamic light scattering data showing the size of aggregates formed by lipid **1** at 2.5 times and 6 times the critical micellar concentration (CMC = 21 mM), and in the presence of octanol droplets, in a solution of lipid at 2.5 times the critical micellar concentration. **d** Average speed of a 50 µm octanol droplet for increasing concentrations of lipids in the reaction medium. Each point is an average of five droplets, and the error bars indicate standard deviation. The droplets move only when the lipid concentration reaches above CPC (~ 28 mM for 50 µm droplets).

Droplet movement is complex and goes through three phases (Fig. 2a and Supplementary Movie 3). At the start, the octanol removed from droplets by micelles forms a corona around the droplet. This corona is a visual signature for octanol-filled micelles that are formed around the droplets (Fig. 2b). In the absence of octanol droplets, dynamic light scattering experiments show that lipid **1** aggregates into objects of ~2.5 nm in diameter. Their diameter remains constant with increasing concentration, which is a typical behavior for micelles. In the presence of octanol, these micelles take up octanol and their diameter consequently increases from ~2.5 nm to ~7.0 nm (Fig. 2c).

### Reciprocal coupling between droplet motion and molecular chemistry

A lag phase separates the moment when the lipid starts forming, from the moment the droplets start moving. The lag phase can be rationalized by the fact that the droplets start moving only once enough micelles are formed, which means that the lipid concentration has to reach a critical propulsion concentration (CPC, Fig. 2d) before movement picks up. The CPC is ~25% larger than the critical micellar concentration CMC (Supplementary Fig. 4), suggesting that the lipids first aggregate around the octanol droplets, and then they form micelles. Once a minimum concentration of lipid micelles is reached, these micelles start drawing octanol away from the droplets and into the aqueous solution. Supplementary Movie 4 and Supplementary Figure 5 show a dyed octanol droplet solubilizing in the lipid-producing system. Octanol-filled micelles are larger than empty micelles (Supplementary Figure 6). Because of this uptake of octanol by lipid micelles, the distribution of the lipids at the droplet interface is disrupted, and a gradient of interfacial tension appears, which makes the droplet move—a process that generally is referred to as Marangoni propulsion^40–46^. When motile, the droplets are away from equilibrium, and eventually they stop once there are no longer enough empty micelles to take up octanol.

**Fig. 3:**
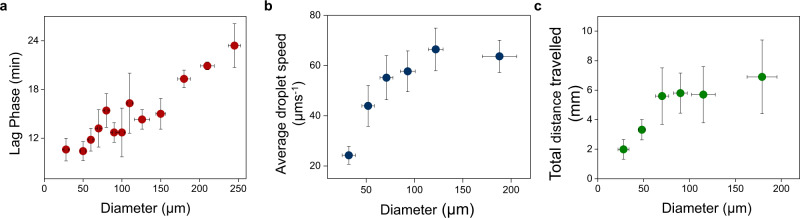
Characteristics of droplet motility from lipid reproduction. **a** Effect of octanol droplet size on the lag phase. Each point is an average of five droplets, and the error bars indicate standard deviation. **b** Average speed of droplets as a function of their diameter. Each point is an average of five droplets, and the error bars indicate standard deviation. **c** Total distance traveled by octanol droplets, as a function of their diameter. Each point is an average of five droplets, and the error bars indicate standard deviation.

In the experimental conditions we use, the duration of the lag phase does not depend on the total number of octanol droplets, because the quantity of lipids produced by the chemical reaction at the critical propulsion concentration is much larger than the amount of lipids required to saturate the droplet interface (see estimations in Supplementary Methods). Note that the lag phase is longer for larger droplets, as higher lipid concentrations are needed to create a gradient of interfacial tension (Fig. 3a).

### The motile behavior of octanol droplets feeds back into the chemical reaction

**Fig. 4:**
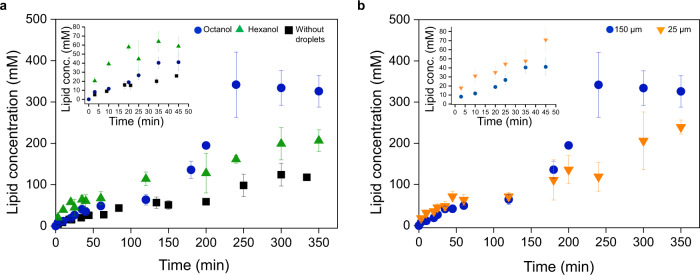
Reciprocity in the coupling between lipid reproduction and droplet motility. **a** Formation of lipid **1** in an oil-in-water emulsion of MPC (1.1 eq.), Cs_2_CO_3_ (0.2 eq.) and hexanethiol (1 eq.). The kinetics are reported in the presence of motile droplets (150 µm, circle), in the presence of stationary droplets of hexanol (~132 µm, triangle) and in the absence of droplets (square). Each point corresponds to an average of three experiments, and the error bars indicate standard deviation. **b** Formation of lipid **1** in the presence of 150 µm octanol droplets (circle) and 25 µm octanol droplets (inverse triangle). Each point corresponds to an average of three experiments, and the error bars indicate standard deviation.

Once the octanol droplets start moving, the bond-forming chemistry leading to the formation of the lipids is significantly accelerated by the motile behavior of the octanol droplets, as evidenced by kinetic investigations on heterogeneous solutions, using ^1^H NMR spectroscopy (Fig. 4 and Supplementary Figs. 7–10). In the presence of motile droplets of octanol, the rate of lipid formation is increased. In the absence of motile droplets, the increase in the rate of lipid formation is not observed. The curve that follows lipid production in the absence of droplets is a sigmoid^21^. The timescale of our experiments corresponds to the beginning of the sigmoidal curve, where the sharp increase in concentration is not yet visible. The effect of droplet motility on the chemical reaction is observed at time scales that are longer than the lifespan of the octanol droplets. A delay between molecular events and their signature at the ensemble level is the very nature of autocatalytic processes –because enough micelles have to be created, before these supramolecular events are translated into a substantial acceleration and sigmoidal response.

The kinetic experiments were also performed in the presence of stationary oil droplets. Indeed, hexanol droplets remain stationary in the reaction mixture over the timescale of the experiment. The size dispersity of these hexanol droplets is larger than with octanol droplets (Supplementary Fig. 3), because their viscosity is lower in comparison to octanol droplets (4.47 mPa.s for hexanol and 7.363 mPa.s for octanol). Hexanol droplets thus dissolve completely before the instability caused by interfacial tension gradients can emerge. Instead, these droplets are taken up rapidly by lipid micelles and disappear after ~20 min, without gaining motility. Figure 4a shows a small increase in reaction rate in the presence of hexanol droplets which we attribute to the added interfacial area that is transiently present in the medium; however, the substantial acceleration induced by motile octanol droplets is not observed. The introduction of hexanol droplets enhances the chemical reactivity immediately by the addition of a fixed amount of interface in the system. This effect is short-lived and has a minor impact on the long-term run of the reaction. Droplets of oils such as mineral oils, silicon oil, and oleic acid, with viscosity higher than 12 mPa.s did not propel. We thus note that viscosity is a key parameter to observe reciprocity and chemo-motile coupling. When the viscosity is too low the droplets disappear before an instability can be established. Droplets of oils with large viscosities are also known not to propel. The range of values of oil viscosity under which we observed the reciprocity between chemistry and movement was between 5 mPa.s and up to 12 mPa.s.

In addition to their viscosity, the size of motile octanol droplets is a key parameter for the coupling between lipid forming reaction and motility, because their size dictates the specifics of their motile behavior, and in particular the average path traveled by the active interface (Fig. 3). Larger droplets (i) start moving later; (ii) move faster (Fig. 3b); and (iii) travel longer distances (Fig. 3c)—which means that they are more effective in bringing active interfaces in reagent-rich areas. In a closed circular chamber, motility of droplets with a diameter of 150 µm starts after a lag phase of 15 min. This starting time for motility cannot be directly extrapolated to the kinetic analysis because these experiments are performed in different experimental set-ups, with different interfacial conditions. Comparing the behavior of systems that include motile droplets of either ~25 µm or ~150 µm in diameter, while keeping the total interfacial area constant, reveals that smaller droplets are indeed less effective in accelerating lipid reproduction (Fig. 4b).

**Fig. 5:**
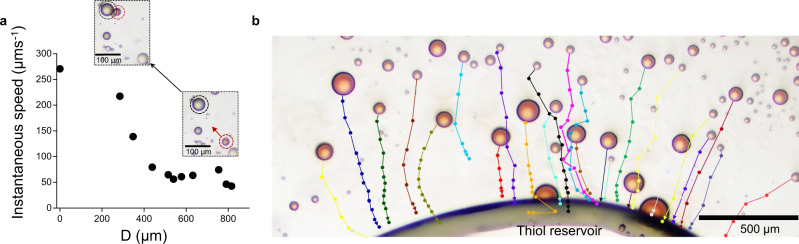
Chemotactic behavior of octanol droplets. **a** Instantaneous speed of an ~50 µm octanol droplet as it moves towards a reservoir of lipid precursor (1-hexanethiol), along a gradient in micellar concentration. The distance between the octanol droplet and the reservoir is noted D. All droplets displayed chemotactic behavior. **b** Chemotactic movement of polydisperse octanol droplets towards a thiol reservoir (Supplementary Movie 5). This experiment was repeated three times independently.

Speed profiles for individual droplets feature peaks (Fig. 2b and Supplementary Fig. 11), where the speed of the droplet increases, likely in the vicinity of areas that are richer in micelles (Fig. 5a). As micelles fuel the movement, the droplets follow lipid micelle concentration gradients—the micelles are more abundant in thiol-rich areas where the lipid is produced. When 1-hexanethiol was present as a large reservoir, rather than dispersed throughout the aqueous phase as an oil-in-water emulsion, octanol droplets demonstrated chemotaxis towards the thiol reservoir (Fig. 5b, Supplementary Movie 5 and Supplementary Movie 6). Chemotactic motion is typically observed with interfacially propelled liquid droplets^36^.

## Discussion

**Fig. 6:**
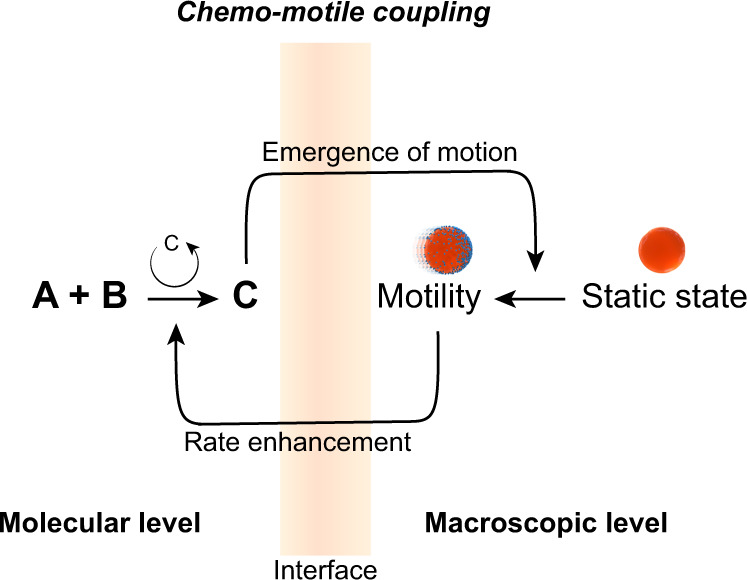
Reciprocal coupling between motility and chemistry across length scales. Self-reproducing lipids initiate and accelerate the movement of oil droplets and, reciprocally, the chemotactic movement of the droplets increases the rate of lipid reproduction.

As the bond-forming chemical reaction causes chemotactic motility of the octanol droplets, in return, the movement of the droplets increases the rate of lipid formation (Fig. 6). Our experimental results allow rationalizing this process: (1) the phase-separated reagents form a lipid, with the lipids self-assembling into micelles that both facilitate the combination of reagents to form lipid, and fuel the motion of octanol droplets. (2) the moving droplets actively explore their heterogeneous environment, and by chemotaxis move to reagent-rich areas, which facilitates the combination of reagents to form even more lipid. Bringing reagents together is particularly important in the kinetics of biphasic reactions^16^ and, in our system, the chemotactic propulsion of motile droplets introduces a form of mixing that allows the hydrophobic and hydrophilic reagents to meet.

In conclusion, we have demonstrated reciprocal coupling between self-reproducing chemistry and microscopic movement, in an entirely artificial system. The mechanism is based on mechanical instabilities, where self-replicating lipids accumulate at the interface of a preformed octanol droplet, until the interface becomes crowded enough that the lipids get depleted by micelles, and as a result the droplet moves forward. The lipid-covered octanol droplets move chemotactically under the effect of lipid self-reproduction. In return, the motile droplets mix the heterogeneous system in which they move, and their chemotactic behavior brings active interfaces in reagent-rich areas, so their motile behavior increases the rate of lipid reproduction. A mutualistic interaction between lipid reproduction and droplet motion is thus established away from equilibrium, and this complex behavior emerges simply by mixing two chemicals in water and octanol. We propose that rate enhancement driven by motile droplets could be generalizable to a variety of other micro-heterogeneous environments, e.g. coacervates and polymer condensates in micellar water. Our findings may also have implications for industrially relevant chemical transformations that involve emulsions.

## Methods

### Motile behavior of octanol droplets

An oil-in-water emulsion was prepared by adding sequentially 2-methacryloyloxyethyl phosphorylcholine, MPC (100 µL, 0.12 mmol, 1.2 M, 1.1 eq.), a solution of Cs_2_CO_3_ (100 µL, 0.02 mmol, 0.2 M, 0.2 eq.) and 1-hexanethiol (15 µL, 0.11 mmol, 1 eq.) to a 4 mL cylindrical vial. After addition, mixing was performed for 3 s at 1*g* with a 1 cm-long stirrer. This reaction medium was introduced into a chamber prepared by attaching a 1 mm-thick silicone film with a circular well of ∼13 mm diameter, on a glass slide. After the octanol droplets were added, the chamber was sealed with a glass cover slip to avoid artifacts from unwanted flows.

For experiments demonstrating the directional movement of octanol droplets towards a thiol reservoir, the procedure involved the preparation of a glass slide where a ≈ 2 mm circular area was functionalized with a solution of trimethoxy(octadecyl)silane in toluene for 1 h (50 µL, 1.1 µmol, 22 mM), to form a hydrophobic patch on the glass slide. The reaction mixture consisting of a solution of MPC (100 µL, 0.12 mmol, 1.2 M, 1.1 eq.) and Cs_2_CO_3_ in water (100 µL, 0.02 mmol, 0.2 M, 0.2 eq.) was added to the chamber and 1-hexanethiol was introduced next. The thiol formed a reservoir on the functionalized area on the glass.

### Preparation of the oil droplets

Octanol droplets were produced in a poly-dimethyl siloxane microfluidic device fabricated by soft-lithography. A microfluidic flow control system (Fluigent) was used to flow octanol, and the aqueous phase with stabilizing surfactants sodium dodecyl sulfate (4 mM) and MPC (300 mM) to the device using appropriate connectors (Precision tips, Nordson EFD) and tubings (Tygon tubing, Cole-Parmer). The size dispersity of the octanol droplets was narrow (Fig. S3a). The low viscosity of hexanol precluded the preparation of hexanol droplets in a microfluidic chip and therefore the droplets were prepared by mechanical agitation.

### Kinetic studies by ^1^H-NMR spectroscopy

The bond-forming reaction was followed by measuring the in situ concentration of lipid **1** with ^1^H-NMR (400 MHz, Bruker). The reaction medium was prepared identically for visualization of the droplet movement (see above). A separate vial was prepared for each time point. When the time required for the reaction was reached, the appropriate vial was quenched with a solution of HCl in D_2_O (100 µL, 0.04 mmol, 0.4 M, 0.37 eq.). Unreacted thiol was extracted from the reaction mixture by addition of 1 mL of hexane and agitation for precisely 20 s at 3000 rpm, after which the organic phase was removed from the vial using a 1 mL syringe. This process was repeated three times to ensure complete extraction of the thiol. Prior to the third extraction, a solution of acetone in D_2_O (600 µL, 0.04 mmol, 67 mM) was added to the aqueous phase, so acetone could be used as an external standard. Concentrations were calculated by integral of lipid peak at 2.47 ppm (2H) relative to that of the acetone peak at 2.22 ppm (6H) (Figs. S7–S10).

When oil droplets were introduced in the system, the same procedure was followed, with the difference that a 3 µL aqueous solution containing a known number of droplets was added to the vial after stirring. For kinetic studies with octanol droplets ~ 20 droplets of 150 µm and ~ 500 droplets of 25 µm in diameter were added so that the total interfacial area was constant.

### Data acquisition and image analysis

Images and videos of droplet movement were recorded using an inverted microscope equipped with a camera (Nikon microscope Eclipse Ts2 with DS-Fi3 camera). A home-made MATLAB script was used to analyze the trajectory and speed of the droplets. The lag phase is defined as the time separating the mixing of the reagents in the vial, from the beginning of droplet movement. The average droplet speed was calculated by measuring the distance over which a droplet moves every second, for the entire duration of the motion.

### Reporting summary

Further information on research design is available in the Nature Research Reporting Summary linked to this article.

## Supplementary information

Supplementary Information

Description of Additional Supplementary Files

Supplementary Movie 1

Supplementary Movie 2

Supplementary Movie 3

Supplementary Movie 4

Supplementary Movie 5

Supplementary Movie 6

Reporting Summary

## Data Availability

Data supporting the findings of this manuscript are available from the corresponding authors upon reasonable request. A reporting summary for this article is available as a Supplementary Information file. Source data are provided with this paper.
