# Supplementary material for: Acceleration of lipid reproduction by emergence of microscopic motion
Source: Nat Commun. 2021 May 19;12:2959. doi: 10.1038/s41467-021-23022-1 (PMC8134444; doi:10.1038/s41467-021-23022-1)
Supplement: Supplementary file 1 — Supplementary Information [file 41467_2021_23022_MOESM1_ESM.pdf]

Supplementary Information

Acceleration of lipid reproduction by emergence of microscopic motion

Dhanya Babu[1], Robert J. H. Scanes[2], Rémi Plamont[1], Alexander Ryabchun[1], Federico Lancia[1], Tibor Kudernac[1], Stephen P. Fletcher[2*], Nathalie Katsonis[1*]

[1]Stratingh Institute for Chemistry, University of Groningen, Nijenborgh 8, 9747 AG Groningen, The Netherlands
[2] Department of Chemistry, Chemistry Research Laboratory, University of Oxford, UK

*Correspondence to: stephen.fletcher@chem.ox.ac.uk, n.h.katsonis@rug.nl

SUPPLEMENTARY METHOD

Synthesis of 1

Lipid **1** was synthesized following a procedure reported by Matsuno and coworkers[1]. MPC (2.5 g, 8.47 mmol, 1 eq) and 1-hexanethiol (1.30 mL, 9.15 mmol, 1.08 eq) were mixed together in ethanol (10 mL), after which N_2 gas was bubbled for 20 min. Diisopropylamine (DIPA, 4 mol %) was added to the solution as a catalyst (Supplementary Figure 1). After stirring for 18 h at room temperature, ethanol was evaporated under vacuum and the residue was washed three times with 10 mL of hexane to remove unreacted 1-hexanthiol. The solid residue was then dissolved in acetone and filtered to remove unreacted MPC, followed by solvent evaporation under vacuum. Finally, the sample was dried under vacuum overnight and 3.4 g of pure **1** was obtained (yield= 95%). The ^{1}H NMR of lipid **1** agreed with earlier reports[2].

Determination of CMC of 1 by fluorescence spectroscopy

A 10 mM stock solution of Nile red (9-(diethylamino)-2-hydroxy-5*H*-benzo[*a*]phenoxazin-5-one) with λexc = 552 nm and λem = 636 nm in DMSO (dimethyl sulfoxide) was prepared to determine the critical micellar concentration (CMC) of lipid **1**[3]. 1 µL of this solution was added to an aqueous solution of lipid **1** at varying concentrations at room temperature, reaching the final volume of 2 mL. The emission fluorescence spectra were analyzed in a Perkin Elmer LS 55 fluorescence spectrometer between 580 nm and 740 nm. The CMC of 20.8 mM was obtained by plotting the lipid concentration against the maximum fluorescence intensity of Nile red (Figure S3). This value was ~16% smaller than what was earlier reported in literature,[1] which we attribute to the use of a different technique to determine CMC.

Control experiments

Movement of droplets. Control experiments were performed where either MPC or 1-hexanethiol were not added to the chamber. In one set of experiments, aqueous solution of MPC (100 µL, 0.12 mmol, 1.2 M, 1.1 eq.) and Cs_2CO_3 (100 µL, 0.02 mmol, 0.2 M, 0.2 eq.) were added sequentially into a 4 mL cylindrical vial and mixed for 3 s at 80 rpm with a 1 cm-long stirrer. After introducing this reaction mixture into a chamber, the octanol droplets were added and the chamber was immediately sealed to avoid any artefacts due to evaporation. The same mixing procedure was performed in the other set of experiments, using a solution of Cs_2CO_3 (100 µL, 0.02 mmol, 0.2 M, 0.2 eq.) and 1-hexanethiol (15 µL, 0.11 mmol, 1 eq.). This reaction mixture was introduced into a chamber, which was sealed after the addition of octanol droplets. In both these cases, the octanol droplets were observed for longer than their typical lag phase and no movement was observed.

Reactivity of octanol with MPC. Aqueous solutions of 2-methacryloyloxyethyl phosphorylcholine, MPC (100 µL, 0.12 mmol, 1.2 M, 1.1 eq.), and Cs_2CO_3 (100 µL, 0.02 mmol, 0.2 M, 0.2 eq.) were added sequentially to a 4 mL cylindrical vial. After addition, they were mixed for 3 s at 80 rpm with a 1 cm-long stirrer, followed by the injection of the 150 µm octanol droplets in solution. After the initial stirring, the reaction was left to proceed for 24 h without stirring. The vial was quenched with a solution of HCl (100 µL, 0.04 mmol, 0.4 M, 0.37 eq.) in D_2O. The work-up extraction from the reaction mixture was performed by addition of 1 mL of hexane and the mixture was agitated for 30 s at 3000 rpm. After phase separation, the organic

phase was removed from the vial using a 1 mL syringe. This process was repeated three times to ensure complete recover of the product. Prior to the third extraction, a solution of acetone in D_2O (600 µL, 0.04 mmol, 67 mM) was added to the remaining aqueous phase, so that acetone could be used as an external standard.

Droplet tracking in videos

Videos of droplet movement were recorded at 5 frames per second using a Nikon camera attached to a Nikon microscope. A home-made MATLAB script was used to extract images every second from the recorded video, and the position of the droplet was determined in every extracted image. The speed of motility was calculated by determining the distance travelled by a droplet between consecutive frames.

Dynamic light scattering measurements

The size of the aggregates formed by lipid **1** was characterized using a Microtrac Nanotrac Wave W3043. Eppendorf safe lock tubes with 600 µL of lipid **1** in water at required concentrations were used for analysis. To study the solubilization of octanol in lipid solubilization, 50 µL of octanol was added to the lipid solution in the tube.

Fluorescence microscopy experiments

The fluorescent dye Nile red was used to visualize the solubilization of octanol by micelles. Briefly, Nile red was added to pure octanol in a concentration of 10µg/ml prior to the production of droplets. Such produced droplets were then added to a chamber with the reactants prepared as mentioned in the methods section – Motion of octanol droplets. Fluorescence imaging was performed in a Nikon Eclipse LV100N POL microscope using filters of the following excitation and emission wavelength cut offs: λexc = 510-560 nm and λem = 580 nm.

Dependence of lag phase on the number of droplets in the chemical system

The diameter of a lipid head was found to be 0.73 nm and agreed with earlier reports[4]. In our chemical system with droplets, for 10 droplets of 100 µm diameter, the number of lipid molecules required to populate the surface of all droplets is defined by $4\pi r_{droplet}/4\pi r_{lipid}$ and it is equal to $6.64x10^{-13}$ mol. 15µL of 1-hexanethiol (1 eq, MW = 118.24, density = 0.832g/ml at 25°C) used in the experimental conditions corresponds to $12.48x10^{-7}$g, which gives 1.05 $x10^{-7}$ mol of lipid produced in the chemical system. This value is much higher than the amount of lipids required to populate the droplets in the chemical system.

SUPPLEMENTARY FIGURES

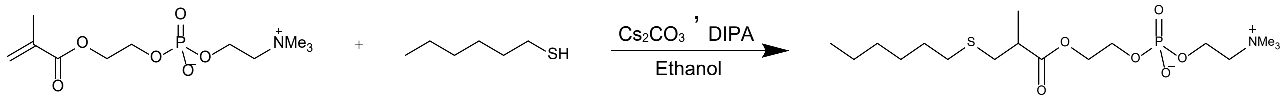

1

Supplementary Figure 1: A Thiol-Michael reaction between hexanethiol and 2-methacryloyloxyethyl phosphorylcholine in aqueous solution to form lipid 1.

a

b

c

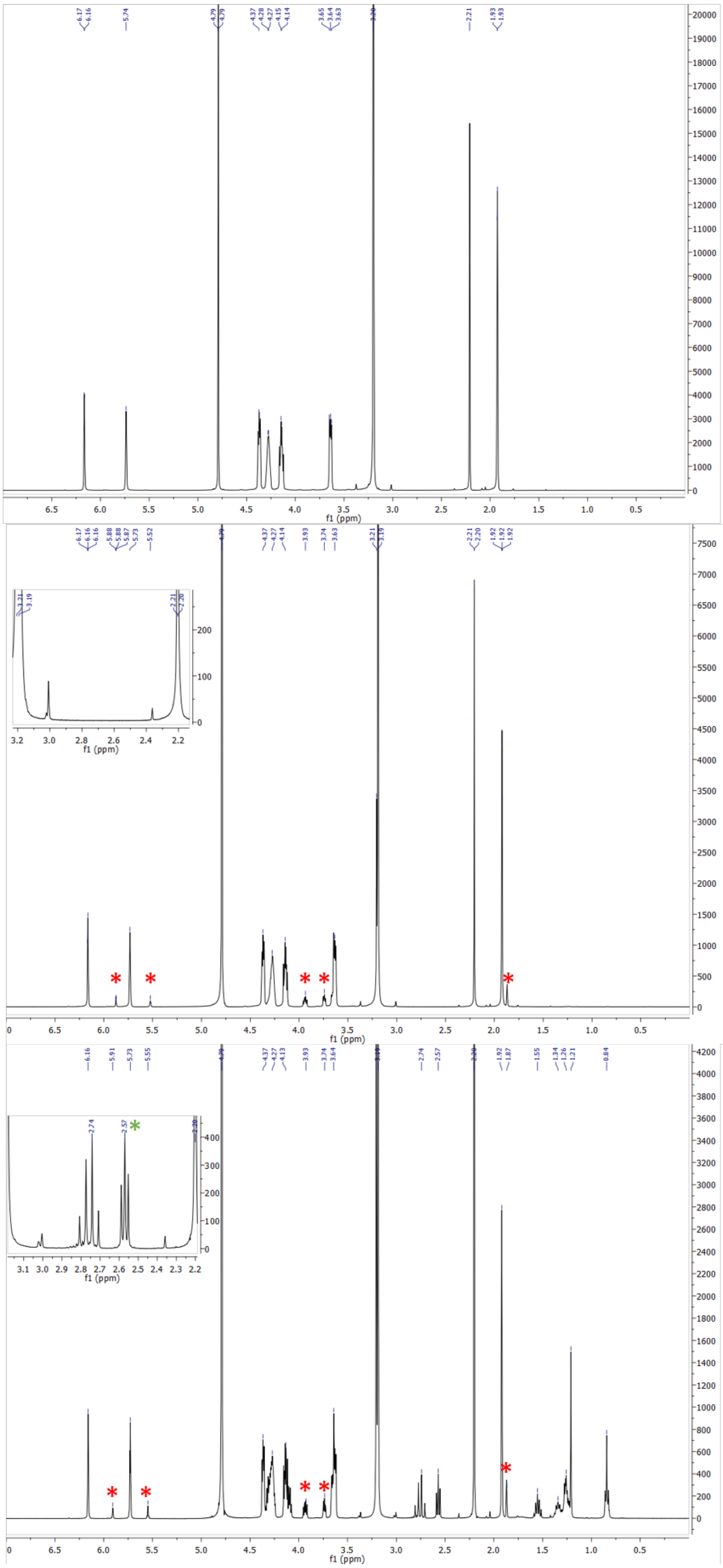

Supplementary Figure 2: Experiment on the reactivity of octanol with MPC. **a**, ^{1}H NMR (400 MHz, D_2O) of MPC with acetone as internal standard. **b**, ^{1}H NMR (400 MHz, D_2O) of the control experiment with zoom on the area where the characteristic peaks of the kinetic study at t= 24h. * indicates product of hydrolysis of MPC in basic conditions. **c**, ^{1}H NMR (400 MHz, D_2O) spectra indicating lipid **1** formed from the reaction of MPC with hexanethiol after 300 min under the reaction conditions used for the kinetic studies. *2.47 ppm is used to follow the concentration of lipid **1**; * indicates the product of hydrolysis of MPC in basic conditions.

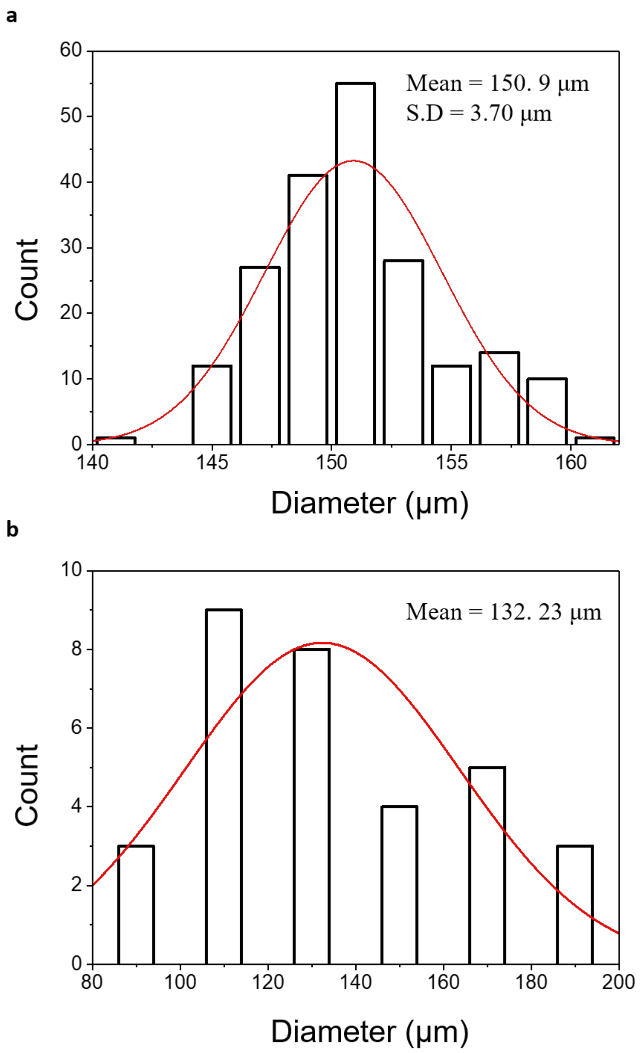

Supplementary Figure 3: Size of the oil droplets used in the study. a, Octanol droplets produced in a microfluidic chip **b**, Hexanol droplets produced by agitation.

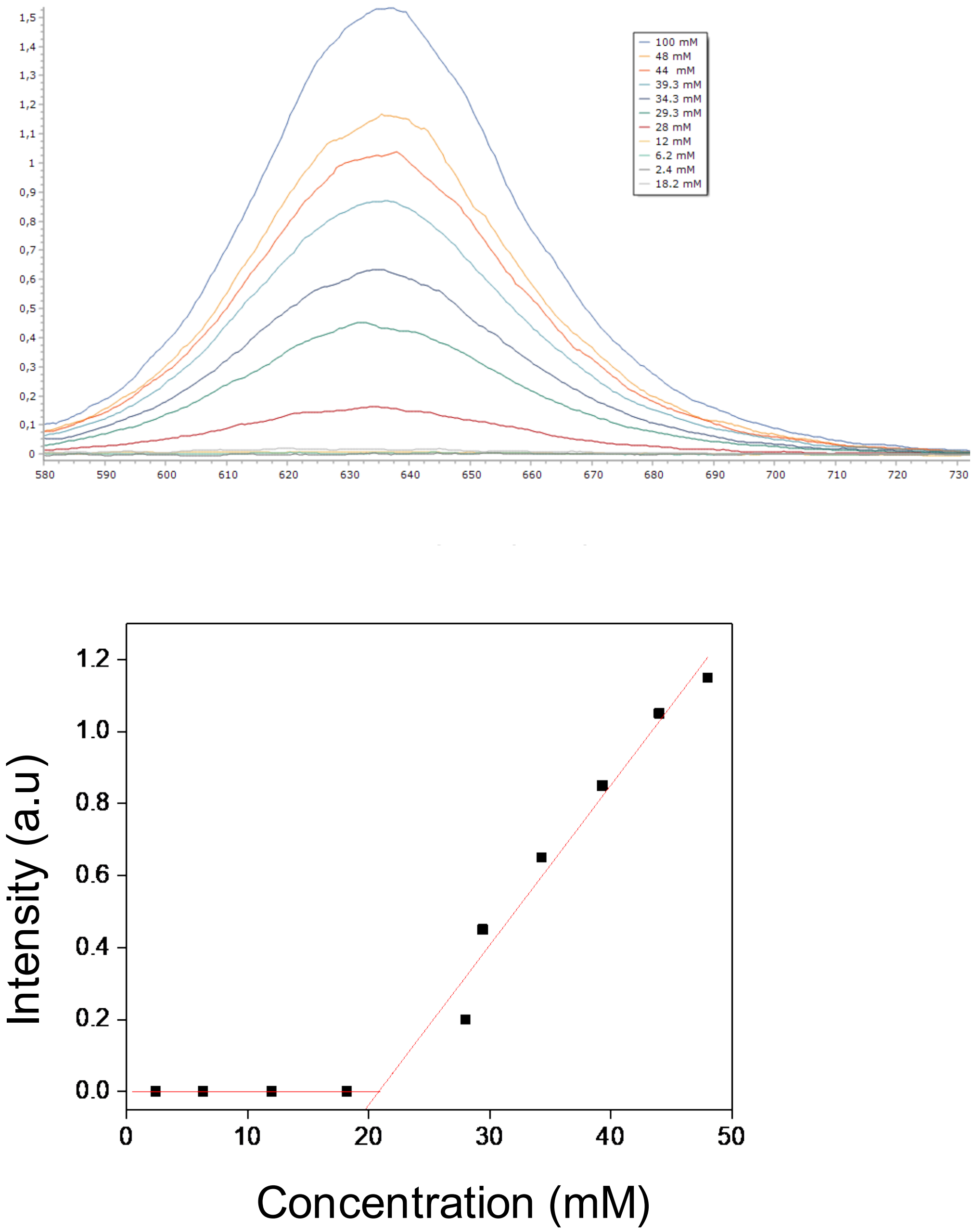

Supplementary Figure 4: Determination of critical micelle concentration of lipid 1. a, stacked fluorescence spectra of aqueous solutions containing Nile Red (10 mM) and lipid **1** at different concentrations. **b**, Maximum fluorescence intensity of Nile Red vs lipid concentration used to obtain the value of the critical micellar concentration.

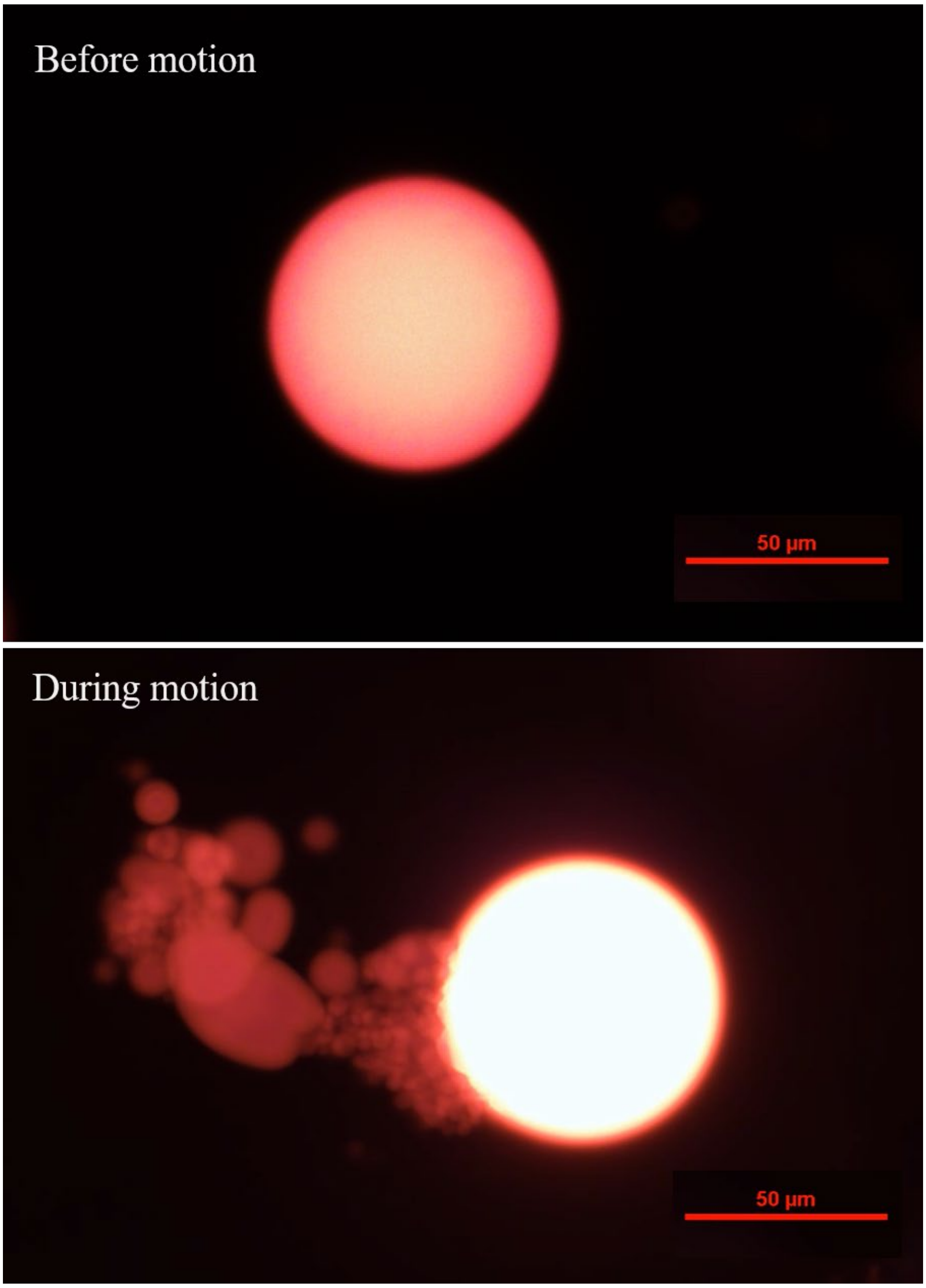

Supplementary Figure 5: Fluorescence microscopy images of an octanol droplet dyed with Nile red, in the chemical system forming lipid 1. Nile red fluorescent dye is added to the octanol droplet to observe its solubilization over time. This experiment was repeated independently three times with similar results.

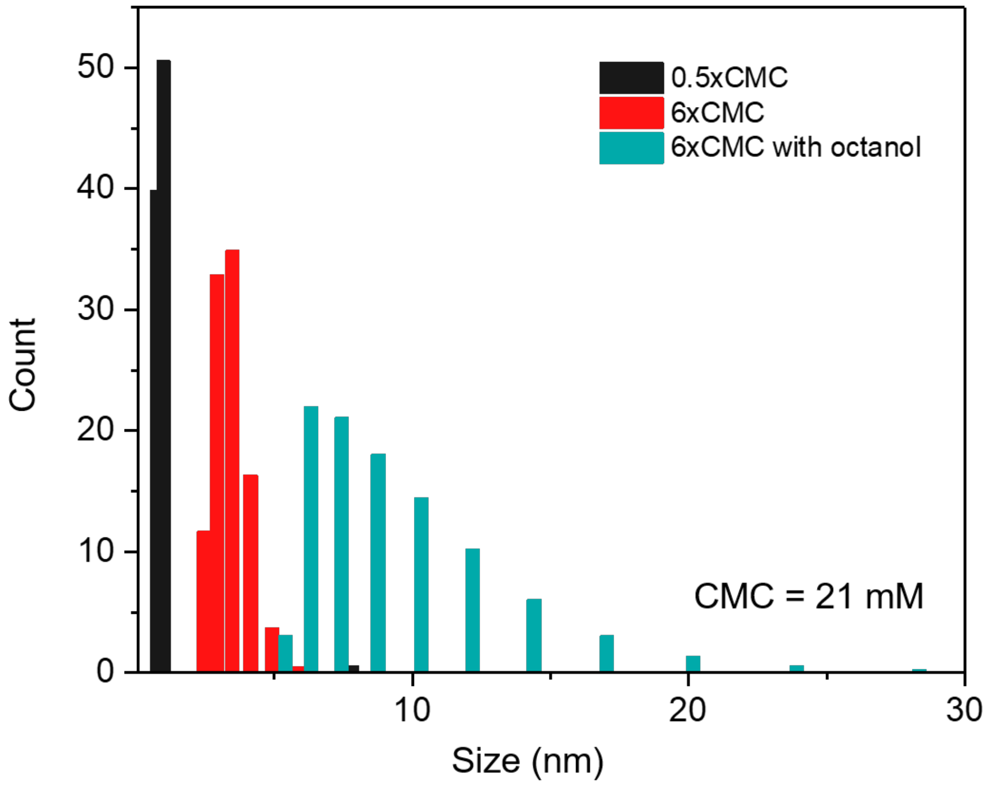

Supplementary Figure 6: Dynamic light scattering data of lipid solution at 0.5xCMC (12mM), 6xCMC (125mM) and 6xCMC in the presence of octanol. The size of lipid aggregates increases in the presence of octanol droplets due to the micellar solubilisation of octanol.

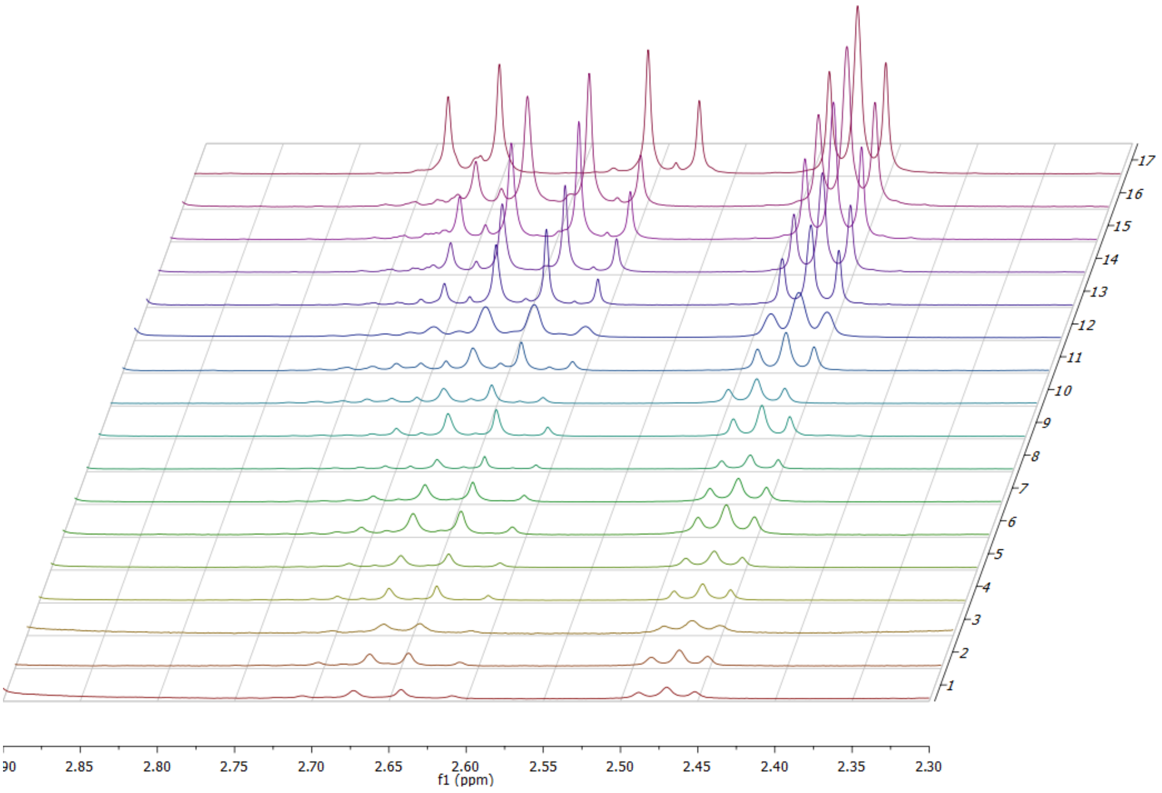

Supplementary Figure 7: Stacked NMR spectra showing the kinetics of formation of lipid 1 in the presence of 150 µm octanol droplets. The concentration of lipid **1** over time, ^{1}H NMR (400 MHz, D_2O) experiments are performed in an oil-in-water emulsion of MPC (1.1 eq.), Cs_2CO_3 (0.2 eq.) and hexanethiol (1 eq.) in the presence of 150 µm octanol droplets. When the required time of the reaction was reached, the appropriate vial was quenched with a solution of HCl in D_2O (100 µL, 0.04 mmol, 0.4 M, 0.37 eq.) Concentrations of the lipid were calculated by comparing the integrated peak at 2.47 ppm to a known concentration of acetone peak at 2.22 ppm used as the internal standard.

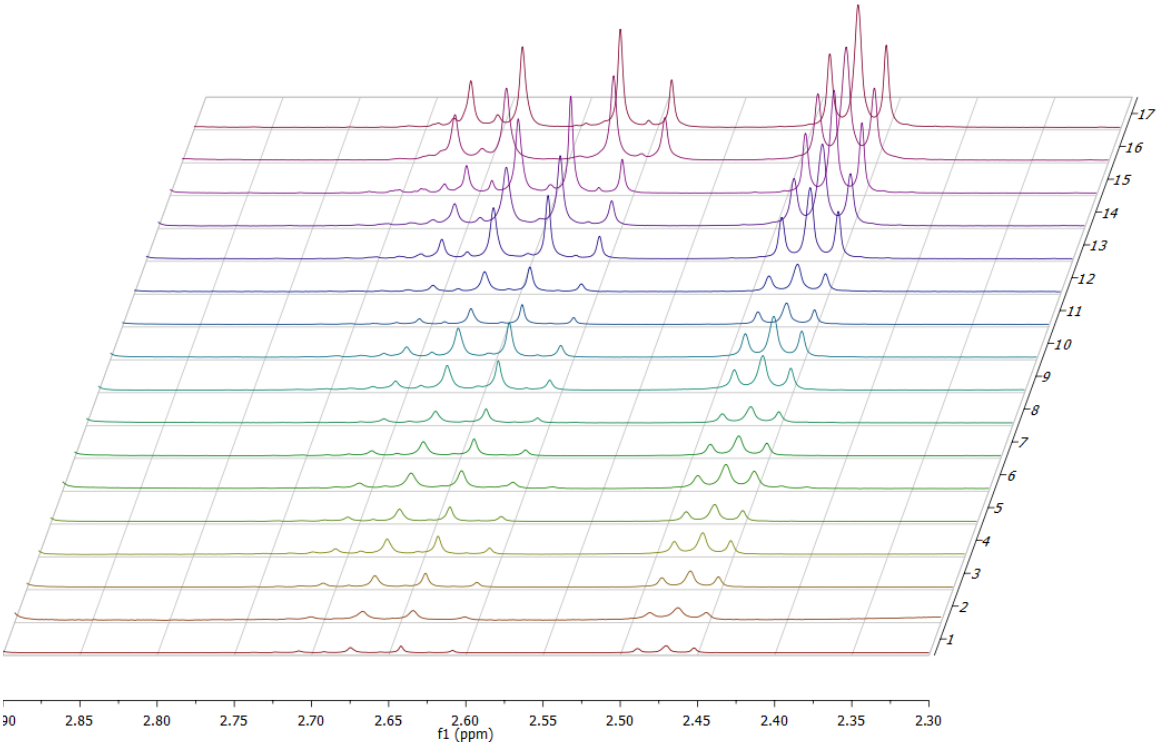

Supplementary Figure 8: Stacked NMR spectra showing the kinetics of formation of lipid 1 in the presence of 25 µm octanol droplets. The concentration of lipid **1** over time, ^{1}H NMR (400 MHz, D_2O) experiments are performed in an oil-in-water emulsion of MPC (1.1 eq.), Cs_2CO_3 (0.2 eq.) and hexanethiol (1 eq.) in the presence of 25 µm octanol droplets. When the required time of the reaction was reached, the appropriate vial was quenched with a solution of HCl in D_2O (100 µL, 0.04 mmol, 0.4 M, 0.37 eq.) Concentrations of the lipid were calculated by comparing the integrated peak at 2.47 ppm to a known concentration of acetone peak at 2.22 ppm used as the internal standard.

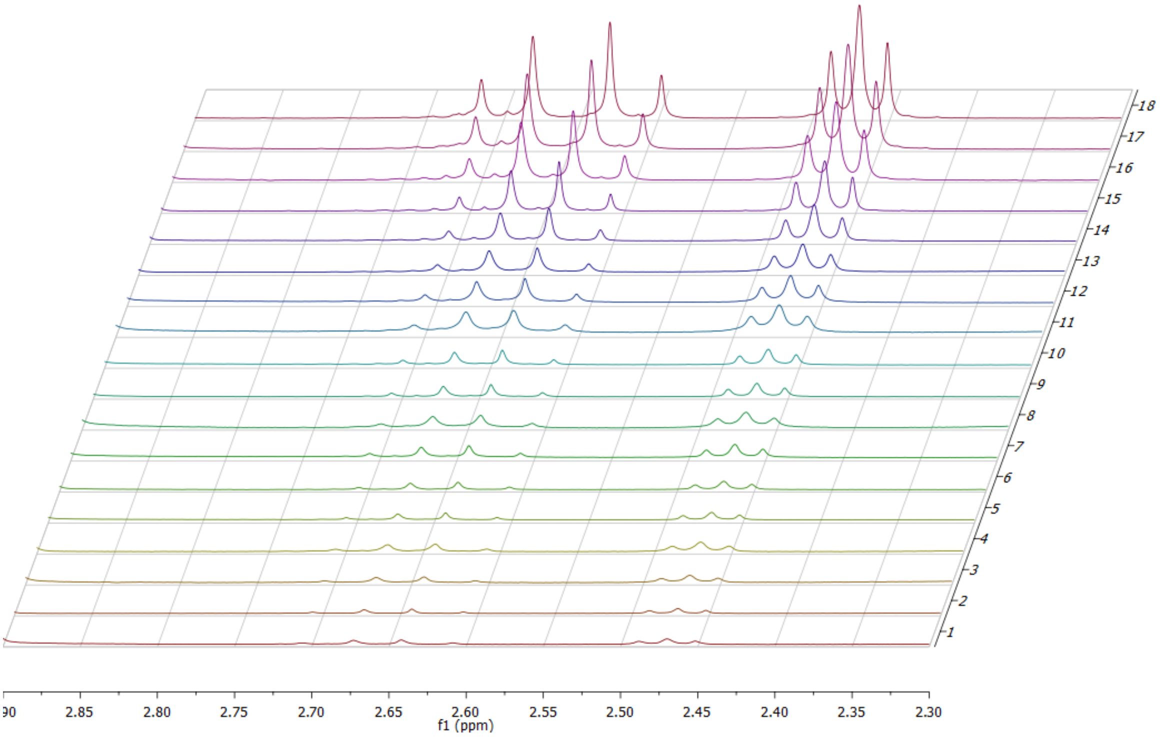

Supplementary Figure 9: Stacked NMR spectra showing the kinetics of formation of lipid 1 in the absence of droplets. The concentration of lipid **1** over time, ^{1}H NMR (400 MHz, D_2O) experiments are performed in an oil-in-water emulsion of MPC (1.1 eq.), Cs_2CO_3 (0.2 eq.) and hexanethiol (1 eq.) in the absence of octanol droplets. When the required time of the reaction was reached, the appropriate vial was quenched with a solution of HCl in D_2O (100 µL, 0.04 mmol, 0.4 M, 0.37 eq.) Concentrations of the lipid were calculated by comparing the integrated peak at 2.47 ppm to a known concentration of acetone peak at 2.22 ppm used as the internal standard.

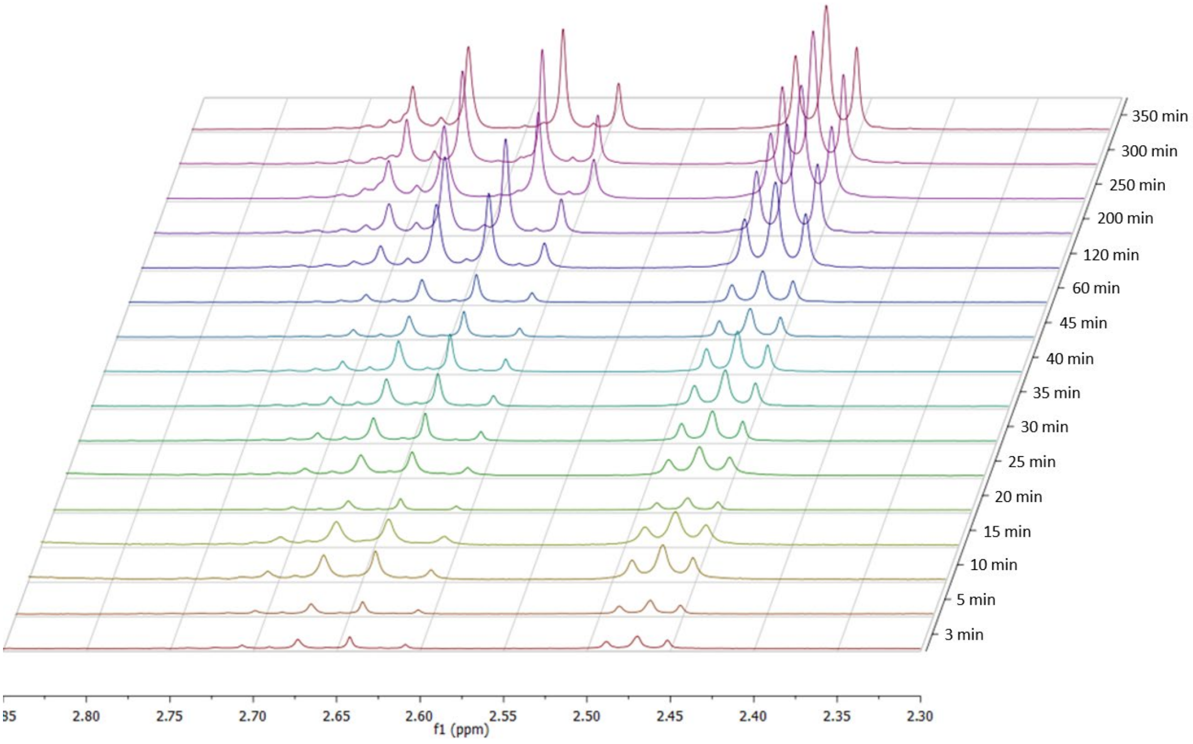

Supplementary Figure 10: Stacked NMR spectra showing the kinetics of formation of lipid 1 in the presence of hexanol droplets. To follow the concentration of lipid **1** over time, ^{1}H NMR (400 MHz, D_2O) are performed in an oil-in-water emulsion of MPC (1.1 eq.), Cs_2CO_3 (0.2 eq.) and hexanethiol (1 eq.) in the presence of hexanol droplets. When the required time of the reaction was reached, the appropriate vial was quenched with a solution of HCl in D_2O (100 µL, 0.04 mmol, 0.4 M, 0.37 eq.) Concentrations of the lipid were calculated by comparison of the integration of the peak at 2.47 ppm to a known concentration of acetone used as an internal standard.

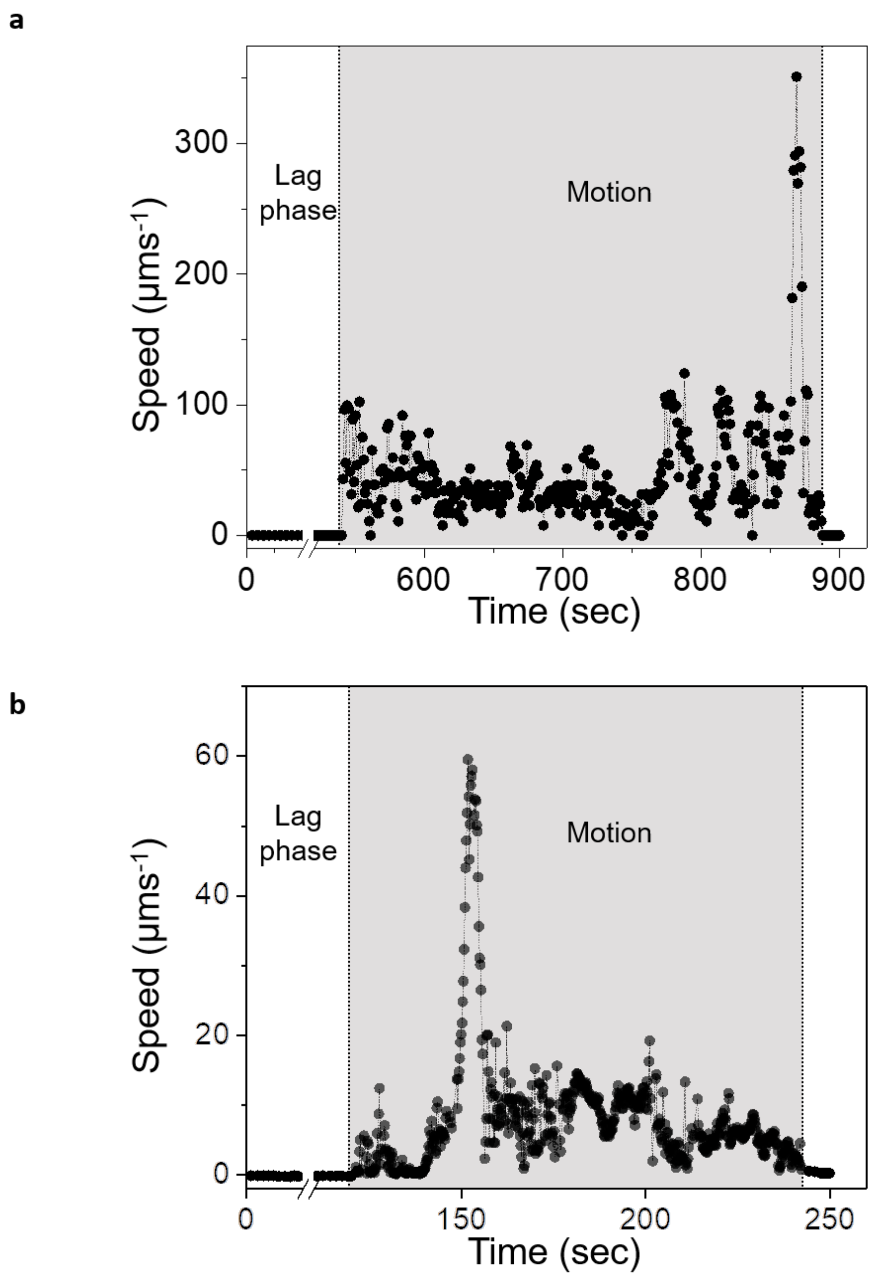

Supplementary Figure 11: Speed of a single octanol droplet over time. a, Speed of a 60 µm octanol droplet (individual trace). **b**, Speed of a 40 µm octanol droplet (individual trace).

SUPPLEMENTARY REFERENCES

1 Matsuno, R., Takami, K. & Ishihara, K. Simple synthesis of a library of zwitterionic surfactants via michael-type addition of methacrylate and alkane thiol compounds. *Langmuir* **26,** 13028–13032 (2010).

2 Bissette, A. J. & Fletcher, S. P. Mechanisms of autocatalysis. *Angew. Chem. Int. Ed.* **52,** 12800–12826 (2013).

3 Chattopadhyay,A.& London, E. Fluorimetric determination of critical micelle concentration avoiding interference from detergent charge. *Anal. Biochem.* **139,** 408–412 (1984).

4 Lipfert, J., Columbus, L., Chu, V. B., Lesley, S. A. & Doniach, S. Size and shape of detergent micelles determined by small-angle X-ray scattering. *J. Phys. Chem. B* **111,** 12427–12438 (2007).
