# Supplementary material for: Acceleration of lipid reproduction by emergence of microscopic motion
Source: Nat Commun. 2021 May 19;12:2959. doi: 10.1038/s41467-021-23022-1 (PMC8134444; doi:10.1038/s41467-021-23022-1)
Supplement: Supplementary file 2 — Description of Additional Supplementary Files [file 41467_2021_23022_MOESM2_ESM.docx]

Description of Additional Supplementary Files

File Name: Supplementary Movie 1.

Description: Production of octanol droplets. The droplets are prepared by microfluidic means, in an aqueous solution containing sodium dodecyl sulfate and MPC. The average size of the droplets is 150 µm (Speed 4x).

File Name: Supplementary Movie 2.

Description: Evidence for a lag phase before droplet motion starts. An octanol droplet (with a red dye) of 250 µm diameter in an aqueous solution containing the precursors of lipid **1**. The movie shows the droplet being solubilized by the lipids that are produced in the system. After a lag phase of 27 min, the droplet leaves behind a corona of micelles and other lipid aggregates (Speed 30x).

File Name: Supplementary Movie 3.

Description: Emergence of microscopic motion from lipid self-reproduction. The video shows the motion of an octanol droplet (100 µm diameter) in an aqueous solution containing the precursors of lipid **1** (Speed 3x).

File Name: Supplementary Movie 4.

Description: Micelle-driven microscopic motility. An octanol droplet (50 µm diameter) dyed with Nile red fluorescent dye is imaged using fluorescence microscopy to observe its solubilization over time in the chemical system (Speed 4x).

File Name: Supplementary Movie 5.

Description: Chemotactic behavior of polydisperse octanol droplets. Directional movement of polydisperse octanol droplets towards a reservoir of thiol (Speed 20x).

File Name: Supplementary Movie 6.

Description: Chemotactic behavior of monodisperse octanol droplets. Directional movement of monodisperse octanol droplets towards a reservoir of thiol (Speed 4x).
